# Measuring levels of osteopontin as potential biomarker for hepatocellular carcinoma in Syrian patients 

**Published:** 2017

**Authors:** Sara Al-Zoubi, Ahmad Wassouf, Almoutassem Billah Zetoune

**Affiliations:** 1 Department of Biochemistry and Microbiology, Faculty of Pharmacy, Damascus University, Damascus, Syria; 2 Department of Internal Diseases, Faculty of Medicine, Damascus University, Damascus, Syria

**Keywords:** Osteopontin, Hepatocellular carcinoma, Chronic liver diseases, Diagnosis, Biomarker

## Abstract

**Aim:**

This study aims to evaluate plasma osteopontin (OPN) levels as a potential biomarker for hepatocellular carcinoma (HCC).

**Background:**

Osteopontin (OPN) is a secreted glycoprotein that is associated with tumorigenesis and metastasis.

**Methods:**

We measured plasma levels of OPN in 26 HCC patients, 27 patients with chronic liver diseases (CLD), and 15 healthy control individuals using a standardized ELISA kit.

**Results:**

The mean plasma OPN level was significantly higher in the HCC group than the CLD group or the normal control group (p-value =0.001/<0.0001). Plasma OPN levels were significantly higher in patients with a tumor size >5 cm in diameter than those with tumors ≤5 cm (p=0.02). OPN levels in the HCC group were not significantly affected by advancing degree of Child-Pugh class. Diagnostic sensitivity and specificity of OPN for HCC were 61% and 82%, respectively (cut-off value: 118.69ng/mL). The area under the ROC curve (AUC) value for OPN was 0.784. However, the AUC value was 0.844 for AFP.

**Conclusion:**

The plasma levels of OPN show low diagnostic accuracy for HCC compared to AFP. However, OPN may have a complementary role in diagnosing HCC in patients with non-diagnostic levels of AFP.

## Introduction

 Hepatocellular carcinoma (HCC) is a primary liver cancer and constitutes one of the most common malignancies worldwide (1). Advances in treatment, imaging, surgical techniques and liver transplantation are a notable step towards better HCC treatment and prevention, and might offer longer survival. Therefore, it is essential to improve prognosis and early HCC detection to fully make use of those treatments (2, 3).

Osteopontin (OPN) is a phosphorylated acidic glycoprotein (4). It is an extracellular matrix (ECM) protein secreted by a variety of cells such as osteoclasts, endothelial cells, epithelial cells, and activated immune cells including macrophages and T cells (5-7). OPN mediates diverse cellular functions like adhesion, migration, and survival of several different cell types, such as regulating and propagating inflammatory responses of macrophages, T-cells, and dendritic cells (8). A large body of evidence showed that OPN contributes to tumorigenesis and metastasis (4, 5); it is also expressed in increased levels by tumor cells of multiple cancer types (10). Values of AFP greater than 20ng/mL are considered abnormal, with values greater than 400ng/mL generally considered diagnostic for HCC. Studies have shown that AFP sensitivity and specificity depend on the cutoff value chosen: the higher the AFP cut-off level, the higher the specificity and the lower the sensitivity (11). Because of its clinicopathological associations with tumor progression and putative mechanisms in gastric and liver cancers, better understanding of the role of OPN in tumorigenesis might help with the diagnosis of HCC (7). In this study, we will compare plasma OPN levels between HCC patients, chronic liver diseases (CLDs) patients and healthy control individuals 

## Methods

**Patients and samples**

This study included three groups: the hepatocellular carcinoma group (HCC group) contained 26 patients who were diagnosed with HCC for the first time. The diagnosis of HCC was based on typical imaging patterns and/or histological examinations conducted according to EASL–EORTC Clinical Practice Guidelines (12). The chronic liver disease group (CLD group) contained 27 patients who were diagnosed in the same hospitals during the same period as the HCC group. Finally, a healthy control group contained 15 individuals who showed no abnormality on laboratory examinations. All samples were collected between March 2014 and February 2015 at Al Assad University Hospital and Al Mouwasat University Hospital. Demographic and clinical data on the etiology of CLD or HCC, the presence of LC, status of liver function in terms of Child-Pugh class, and tumor-nodes-metastases (TNM) stage of HCC were determined according to the AJCC staging criteria (13, 14), and the Barcelona Clinic Liver Cancer (BCLC) stage (12) was determined by reviewing medical records and radiological studies. Serum AFP levels were routinely evaluated in all patients. In all samples, 5 mL of blood was collected in an ethylene diamine tetra acetic acid (EDTA) plastic tube, and isolated plasma samples were stored at −80◦C until measurements of OPN. The study was approved by the ethical commission of Damascus University, and written informed consent was obtained from all patients when they were enrolled. Measurement of Plasma OPN level: Plasma OPN levels were measured using an enzyme-linked immune sorbent assay (ELISA) kit (Osteopontin Human ELISA Kit (ab100618)) according to the manufacturer’s instructions.

**Statistical analysis**

Means, ranges and standard deviations were used for descriptive statistics. The difference in OPN concentrations between the three groups, as well as the association of plasma OPN with tumor staging, was evaluated using the Kruskal-Wallis H test. The difference in OPN concentrations between the two different groups, and the association of plasma OPN with other variables were evaluated with the Mann–Whitney U test. Correlation between plasma levels of OPN and AFP were analyzed using Spearman’s correlation coefficient. ROC Curve analysis was used to evaluate the diagnostic value of each tumor marker. The optimal cutoff values were calculated using the maximum sum of sensitivity and specificity. Statistical analyses were conducted using IBM SPSS Statistics 20 and Microsoft Excel 2010. 

## Results

**Figure 1:**
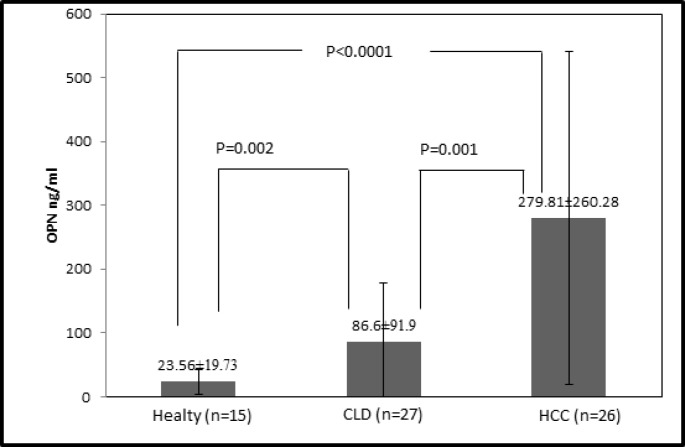
Plasma levels of Osteopontin in hepatocellular carcinoma (HCC) group, chronic liver disease (CLD) group and healthy group. The median plasma OPN level in HCC was significantly higher than in CLD and healthy controls (p-value =0.001/<0.0001 by Mann-Whitney U test

The demographic characteristics of patients included in the analysis are summarized in Table 1. The plasma OPN levels of the HCC group, the CLD group and the normal healthy control group are shown in Figure 1. 

**Table 1:** Clinical characteristics of study patients (n=68

Characteristic	HCC group (n=26)	CLD group (n=27)	Control group (n=15)
Age (year)			
≤50	5 (19%)	13 (48%)	6 (40%)
>50	21 (81%)	14 (52%)	9 (60%)
Sex			
Male	20 (77%)	13 (48%)	4 (27%)
Female	6 (23%)	14 (52%)	11 (73%)
Tumor size (cm)			
≤5	16 (62%)		
>5	10 (38%)		
Etiology			
HBV	10 (39%)	14 (52%)	
HCV	5 (19%)	2 (7%)	
Cirrhosis without viral infection	7 (27%)	7 (26%)	
Unknown cause	4 (15%)	4 (15%)	
Presence of cirrhosis	14 (54%)	20 (74%)	
Child-Pugh class A/B/C	-/8/18		
TNM stage I/II/III/IV	4/8/11/3		
BCLC stage A/B/C/D	2/3/3/18		

Data is presented as number (%). HCC: hepatocellular carcinoma, CLD: chronic liver disease, HBV: hepatitis B virus, HCV: hepatitis C virus.

The mean (range) plasma OPN level in HCC, CLD and healthy controls were 279.81 (890.77-29.3), 86.6 (368.74-7.12), 23.56 (63-0.99) ng/mL, respectively. The mean plasma OPN level in the HCC group was significantly higher than the CLD and healthy controls (p-value =0.001/<0.0001 by Mann-Whitney U test). Table 2 shows OPN levels in the HCC group based on various clinical parameters. Plasma OPN levels in the HCC group were not significantly affected by sex, etiology of HCC (HBV), Child-Pugh class or the tumor stage. However, plasma OPN levels were significantly lower in patients aged 50 years or below compared to those above 50 [68.55 (29.3-157.21) ng/mL vs 330.11 (43.91-890.77) ng/mL, p=0.012]. The presence of cirrhosis was negatively associated with plasma OPN levels [172.13 (35.2-466.83) ng/mL vs 405.44 (29.3-890.77) ng/mL, p=0.024]. Plasma OPN levels were significantly higher in patients with tumor size >5 cm in diameter than patients with ≤5 cm tumor size [423.05 (91.85-890.77) ng/mL vs 190.28 (29.3-560.2) ng/mL, p=0.02].

**Table 2:** Relationship between plasma OPN levels and clinicopathological characteristics of patients with hepatocellular carcinoma

Characteristics	n	Plasma OPN level ng/ml (mean-range)	P-value
Age (year)			0.012
≤50	5	68.55 (29.3-157.21)	
>50	21	330.11 (43.91-890.77)	
Sex			0.465
Male	20	256.95 (29.3-710.98)	
Female	6	356.02 (84.55-890.77)	
Etiology			0.815
HBV	10	249.33 (43.91-710.98)	
Non-HBV	16	302.16 (29.3-890.77)	
Liver cirrhosis			0.024
Presence	14	172.13 (35.2-466.83)	
Absence	12	405.44 (29.3-890.77)	
Tumor size (cm)			0.02
≤5	16	190.28 (29.3-560.2)	
>5	10	423.05 (91.85-890.77)	
Child-Pugh class			0.291
B	8	221.93 (29.3-890.77)	
C	18	305.53 (43.91-785.53)	
TNM stage			0.877
I/II	12	255.71 (29.3-560.2)	
III/IV	14	300.47 (43.91-890.77)	
BCLC stage			0.47
A/B	5	122.43 (29.3-343.83)	
C/D	21	371.28 (43.91-890.77)	

P-Value was calculated using Mann-Whitney U test

**Figure 2:**
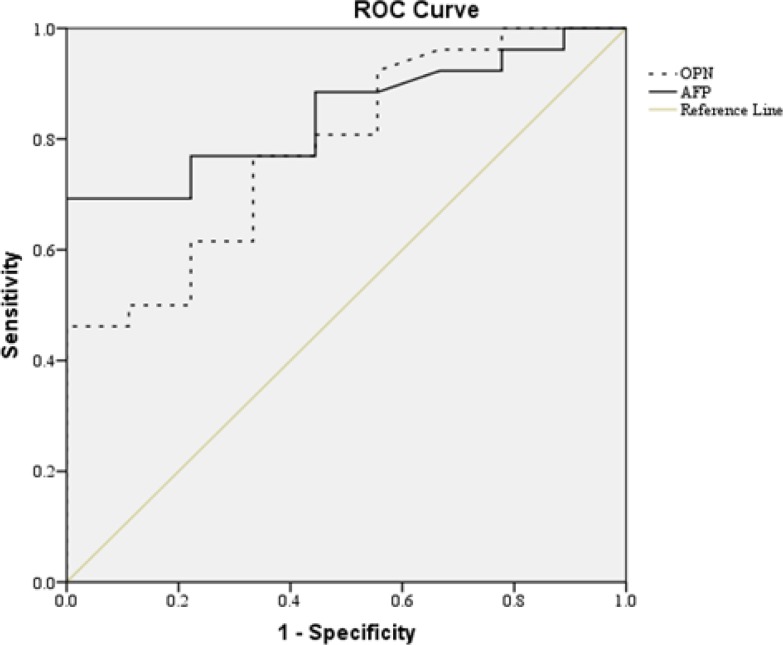
ROC analysis of plasma Osteopontin (OPN) in comparison with α-fetoprotein (AFP). The Area under the curve (AUC) was 0.784 for OPN and 0.844 for AFP

Table 3 shows the sensitivity and specificity of plasma OPN and AFP levels at selected cut-off values, differentiating HCC cases from CLD cases. According to ROC Analysis, the sensitivity and specificity of plasma OPN levels in HCC patients relative to the CLD group were 61% and 82%, respectively, at a cutoff value of 118 ng/mL. For differentiation between HCC and the healthy control group, plasma OPN showed a sensitivity of 80% and a specificity of 100% at a cutoff level of 65.05 ng/mL. Moreover, the sensitivity and specificity of OPN for selective detection of the HCC group over the non-HCC group (CLD group + healthy control group) were 69% and 84%, respectively, at a cutoff level of 102.33 ng/mL. Figure 2 compares the ROC curve for plasma OPN and AFP. The area under the curve (AUC) for plasma OPN was 0.784 (95% P=0.001), while AUC for AFP was 0.844 (95% P=0.002). The correlation between concurrently measured AFP levels and OPN levels was insignificant according to the Spearman rank correlation test (P=0.098). 

**Table 3:** Sensitivity and Specificity of plasma OPN and AFP levels for Hepatocellular Carcinoma and Chronic Liver Disease

	Cutoffs(ng/ml)	Sensitivity (%)	Specificity (%)
AFP	400	53	100
OPN	118.69	61	82

AFP: alpha-fetoprotein. OPN: osteopontin.

## Discussion

In our study, we found that OPN plasma levels were significantly higher in HCC patients than those with chronic liver diseases or healthy controls. OPN levels correlated with age, cirrhosis and size of tumor. In addition, the diagnostic accuracies of OPN were not superior to accuracies for AFP in terms of the AUC. The correlation between OPN and AFP levels was not significant.

Recently, many studies have indicated that increased OPN expression is associated with tumor invasion, progression, or metastasis in many kinds of human cancers, and HCC is no exception (4, 7, 15-17). However, the role of OPN in tumor development is complex and may be affected by many factors. OPN is a notable potential tumor marker, because it is secreted into various kinds of body fluids, including blood, urine and breast milk (15, 18). Previous studies have shown that in HCC, OPN expression is mostly found in malignant hepatocytes and cancer-infiltrating macrophages, not in noncancerous hepatocytes or Kupffer cells. This strongly suggests that the source of elevated plasma OPN in HCC is the cancer tissue itself (4, 5). Our data showed that plasma OPN levels were elevated in HCC patients. It also showed that sex, etiology of HCC (HBV) or tumor stage were not significantly associated with plasma OPN levels. In HCC patients, OPN levels were negatively associated with the presence of cirrhosis. A similar result has been reported by Lee in 2014 (19). This confirms the relevance of increased plasma OPN levels to the process of carcinogenesis rather than cirrhosis. Our results revealed that the plasma OPN level in HCC patients with a tumor >5cm in diameter was significantly higher compared to patients with tumors ≤5cm in diameter. A similar result was reported by Sun in 2009 (16). None of the HCC patients with a tumor >5cm in diameter had cirrhosis. Further studies are needed to confirm this finding. The sensitivity and specificity of plasma OPN for HCC differentiation from CLD were 61% and 82%, respectively, at a cutoff value of 118.6 ng/mL. The AUC of plasma OPN and AFP were 0.784 and 0.844, respectively, which suggests that determining OPN levels might not be superior to analyzing plasma levels in terms of the diagnostic efficacy for HCC. AFP levels above 400 ng/mL are widely accepted as a diagnostic for HCC. Interestingly, 13 out of 26 patients with HCC had AFP levels under 400 ng/mL. When plasma OPN levels were measures in these patients, the diagnostic efficacy of OPN was similar to the results of total HCC patients, regardless of AFP level. The correlation between OPN and AFP levels was not significant. Therefore, plasma OPN levels might be helpful for the diagnosis of HCC in the patients with non-diagnostic AFP levels. This finding was in agreement with the results of Kim in 2006, who found that some HCC patients had AFP levels under 400 ng/mL, while having high plasma OPN levels (5). This study had a limitation with the methodology used. We measured levels of OPN using stored plasma samples, but levels of AFP were measured using samples taken for routine laboratory examinations. Ideally, all biomarkers should have been measured using the same samples. In conclusion, our study showed that plasma OPN levels were significantly elevated in patients with HCC compared to those with CLD. Furthermore, plasma OPN levels had low diagnostic accuracy for HCC compared with the accuracy achieved with AFP. However, OPN may have a complementary role in diagnosing HCC in patients with low levels of AFP. The ultimate diagnostic utility and implication of plasma OPN in HCC will be determined in future studies.

## Conflict of interest

 The authors declare that they have no conflict of interest.

## References

[1] Davis GL, Dempster J, Meler JD, Orr DW, Walberg MW, Brown B (2008). Hepatocellular carcinoma: management of an increasingly common problem. Bayl Univ Med Cent.

[2] Yang JD, Roberts LR (2010). Hepatocellular carcinoma: a global view. Nat Rev Gastroenterol Hepatol.

[3] Colombo M, Sangiovanni A (2015). Treatment of hepatocellular carcinoma: beyond international guidelines. Liver Int.

[4] Qin L (2014). Osteopontin is a promoter for hepatocellular carcinoma metastasis: a summary of 10 years of studies. Front Med.

[5] Kim J, Ki SS, Lee SD, Han CJ, Kim YC, Park SH (2006). Elevated Plasma Osteopontin Levels in Patients with Hepatocellular Carcinoma. Am J Gastroenterol.

[6] Lou X, Ruhland MK, Pazolli E, Lind AC, Stewart SA (2011). Through Activation of the MAPK Pathway Osteopontin Stimulates Preneoplastic Cellular Proliferation. Mol Cancer Res.

[7] Cao DX, Li ZJ, Jiang XO, Lum YL, Khin E, Lee NP (2012). Osteopontin as potential biomarker and therapeutic target in gastric and liver cancers. World J Gastroenterol.

[8] Lund SA, Giachelli CM, Scatena M (2009). The role of osteopontin in inflammatory processes. J Cell Commun Signal.

[9] Rodriguse LR, Teixeira JA, Schmitt FL, Paulsson M, Lindmark-Mansson H (2007). The Role of Osteopontin in Tumor Progression and Metastasis in Breast Cancer. Cancer Epidemiol Biomarkers Prev.

[10] Shevde LA, Samant RS (2014). Role of osteopontin in the pathophysiology of cancer. Matrix Biology.

[11] Daniele B, Bencivenga A, Megna AS, Tinessa V (2004). Alpha-fetoprotein and ultrasonography screening for hepatocellular carcinoma. Gastroenterology.

[12] Llovet JM, Ducreux M, Lencioni R, Di Bisceglie AM, Galle PR, Dufour JF (2012). EASL–EORTC Clinical Practice Guidelines: Management of hepatocellular carcinoma. Journal Of Hepatology.

[13] Bruix J, Sherman M (2011). Management of Hepatocellular Carcinoma: An Update. HEPATOLOGY.

[14] Jelic S, Sotiropoulos GC (2010). Hepatocellular carcinoma: ESMO Clinical Practice Guidelines for diagnosis, treatment and follow-up. Annals of Oncology.

[15] Zhang H, Ye QH, Ren N, Zhao L, Wang YF, Wu X (2006). The prognostic significance of preoperative plasma levels of osteopontin in patients with hepatocellular carcinoma. J Cancer Res Clin Oncol.

[16] Sun J, Xu HM, Zhou HJ, Dong QZ, Zhao Y, Fu LY (2010). The prognostic significance of preoperative plasma levels of osteopontin in patients with TNM stage-I of hepatocellular carcinoma. J Cancer Res Clin Oncol.

[17] Ramaiah SK, Rittling S (2008). Pathophysiological Role of Osteopontin in Hepatic Inflammation, Toxicity, and Cancer. TOXICOLOGICAL SCIENCES.

[18] Abu El Makarem MA, Abdel-Aleem A, Ali A, Saber R, Shatat M, Abdel Rahem D (2011). Diagnostic significance of plasma osteopontin in hepatitis C virus-related hepatocellular carcinoma. ANNALS of Hepatology.

[19] Lee HJ, Yeon JE, Suh SJ, Lee SJ, Yoon EL, Kang K (2014). Clinical Utility of Plasma Glypican-3 and Osteopontin as Biomarkers of Hepatocellular Carcinoma. Gut and Liver.

